# Septaly Oriented Mild Aortic Regurgitant Jets Negatively Influence Left Ventricular Blood Flow—Insights From 4D Flow MRI Animal Study

**DOI:** 10.3389/fcvm.2021.711099

**Published:** 2021-08-09

**Authors:** Nikola Cesarovic, Miriam Weisskopf, Mareike Kron, Lukas Glaus, Eva S. Peper, Stefano Buoso, Simon Suendermann, Marko Canic, Volkmar Falk, Sebastian Kozerke, Maximilian Y. Emmert, Christian T. Stoeck

**Affiliations:** ^1^Department of Health Sciences and Technology, Swiss Federal Institute of Technology, Zurich, Switzerland; ^2^Department of Cardiothoracic and Vascular Surgery, German Heart Center Berlin, Berlin, Germany; ^3^Division of Surgical Research, University Hospital Zurich, University of Zurich, Zurich, Switzerland; ^4^Institute for Biomedical Engineering, University and ETH Zürich, Zurich, Switzerland; ^5^Department of Cardiovascular Surgery, Charité-Universitätsmedizin Berlin, Berlin, Germany; ^6^Institute for Regenerative Medicine, University of Zurich, Zurich, Switzerland

**Keywords:** paravalvular leakage, 4D flow MRI, vortex formation, intraventricular hemodynamics, aortic regurgitation, mild regurgitation, translational large animal model

## Abstract

**Objectives:** Paravalvular leakage (PVL) and eccentric aortic regurgitation remain a major clinical concern in patients receiving transcatheter aortic valve replacement (TAVR), and regurgitant volume remains the main readout parameter in clinical assessment. In this work we investigate the effect of jet origin and trajectory of mild aortic regurgitation on left ventricular hemodynamics in a porcine model.

**Methods:** A pig model of mild aortic regurgitation/PVL was established by transcatheter piercing and dilating the non-coronary (NCC) or right coronary cusp (RCC) of the aortic valve close to the valve annulus. The interaction between regurgitant blood and LV hemodynamics was assessed by 4D flow cardiovascular MRI.

**Results:** Six RCC, six NCC, and two control animals were included in the study and with one dropout in the NCC group, the success rate of model creation was 93%. Regurgitant jets originating from NCC were directed along the ventricular side of the anterior mitral leaflet and integrated well into the diastolic vortex forming in the left ventricular outflow tract. However, jets from the RCC were orientated along the septum colliding with flow within the vortex, and progressing down to the apex. As a consequence, the presence as well as the area of the vortex was reduced at the site of impact compared to the NCC group. Impairment of vortex formation was localized to the area of impact and not the entire vortex ring. Blood from the NCC jet was largely ejected during the following systole, whereas ejection of large portion of RCC blood was protracted.

**Conclusions:** Even for mild regurgitation, origin and trajectory of the regurgitant jet does cause a different effect on LV hemodynamics. Septaly oriented jets originating from RCC collide with the diastolic vortex, reduce its size, and reach the apical region of the left ventricle where blood resides extendedly. Hence, RCC jets display hemodynamic features which may have a potential negative impact on the long-term burden to the heart.

## Introduction

Beside aortic leaflet prolapse and perforation, eccentric aortic valve regurgitation could be caused by paravalvular leakage (PVL) following surgical but more often transcatheter aortic valve replacement. PVL remains a major concern with TAVR and is associated with post-procedural complication created by insufficient sealing between prosthetic valve and the aortic wall. In the past it has been reported that up to 60% of the patients receiving TAVR displayed signs of at least mild aortic regurgitation caused by post-procedural PVL (1). While clinical outcomes and procedural success steadily improved over recent years, still significant regurgitation is observed in more than 17% of cases (2). Presence of moderate/severe aortic regurgitation following TAVR is associated with worse long-term outcomes, impeded reverse remodeling, increased morbidity, and mortality (2–5). Although recent procedural efforts, such as improved prosthesis alignment and commissural overlap (6) as well as adapted implant designs (7), might reduce the likelihood of PVL, the patient pool requiring TAVR has increased considerably, leaving a significant number of patients with this problematic issue (1, 8).

In clinical assessment, regurgitant volume remains the main reference value to determine prognosis and guide therapy in patients with incompetent aortic valve. In cases of severe regurgitation, guidelines give clear therapeutic recommendations (9). However, for mild-to-moderate aortic insufficiency and/or PVL, the situation is less clear. Although it has been shown that moderate or greater PVL induced volume overload following TAVR in patients with hypertrophic left ventricle is one of the causes for increased long-term mortality (2, 4, 10, 11), the effects of the jets origin and trajectory on left ventricular (LV) hemodynamics have not been investigated yet.

Diastolic filling of the left ventricle (LV) is associated with characteristic, vorticial patterns of blood flow (12). These patterns are closely dependent on pressure differences between the cardiac chambers, as well as on the shape and motion of the ventricular walls, valves, and great vessels. Therefore, blood flow patterns have been proposed as a sensitive marker of cardiac health and as a key component of the function itself (13). In pathologies such as myocardial infarction, dilated cardiomyopathy, heart failure, or aortic regurgitations, vortices are disturbed (14–16). Deviations from physiologic diastolic blood flow have also been observed after surgical interventions, creating regurgitant jets at the outer bound of implanted artificial valve (17, 18). In the presence of aortic PVL, turbulent blood flow is reported due to the regurgitant jet, resulting in an increased energy dissipation within the LV (17). As a consequence, the LV not only needs to overcome the volume overload but also requires it to produce additional work (14). Studies employing mathematical models, *in-silico* ventricular models, as well as isolated beating heart experiments clearly demonstrated the effects of significant aortic regurgitant flow on LV energy loss and diastolic vortex formation, highlighting the importance of a more in-depth understanding of its impact on LV myocardial remodeling (18–20).

We hypothesize that in the setting of eccentric mild aortic regurgitation/PVL, not the regurgitant volume *per se*, but rather the regurgitant jet origin and its path within the ventricle affects the diastolic intra-ventricular blood flow with the potential of becoming a predictor or even a catalyst for adverse remodeling and poor clinical outcome.

In this study, we aim to investigate the effects of mild aortic regurgitant/PVL jets originating either from the annular region of either the non-coronary (NCC) or the right coronary cusp (RCC), on diastolic vortex parameters, kinetic energy, and blood residence time. For this purpose, we developed a translational large animal model of mild aortic regurgitation by minimally invasive (transcatheter) piercing of the aortic valve annulus/leaflets and subsequent state of the art cardiovascular magnetic resonance four-dimensional phase contrast flow imaging (4D-flow MRI).

## Materials and Methods

### Animal Protocol

The animal housing and experimental protocols were approved by the Cantonal Veterinary Office, Zurich, Switzerland, under License ZH 213/2019 and ZH 219/2016, and were performed in accordance with Swiss animal protection law and ordinance. Animal housing and experimental procedures also conformed to the European Directive 2010/63/EU of the European Parliament and the Council of September 22, 2010, on the Protection of Animals Used for Scientific Purposes and to the Guide for the Care and Use of Laboratory Animals. A total of 14 female animals (Swiss large white race, 65 ± 5 kg) were included in this study and split into the following groups: NCC PVL *N* = 6, RCC PVL *N* = 6, and Control *N* = 2. Procedure of PVL creation failed in 1 animal in the NCC group, and hence this animal was excluded from the study.

**Figure 1:**
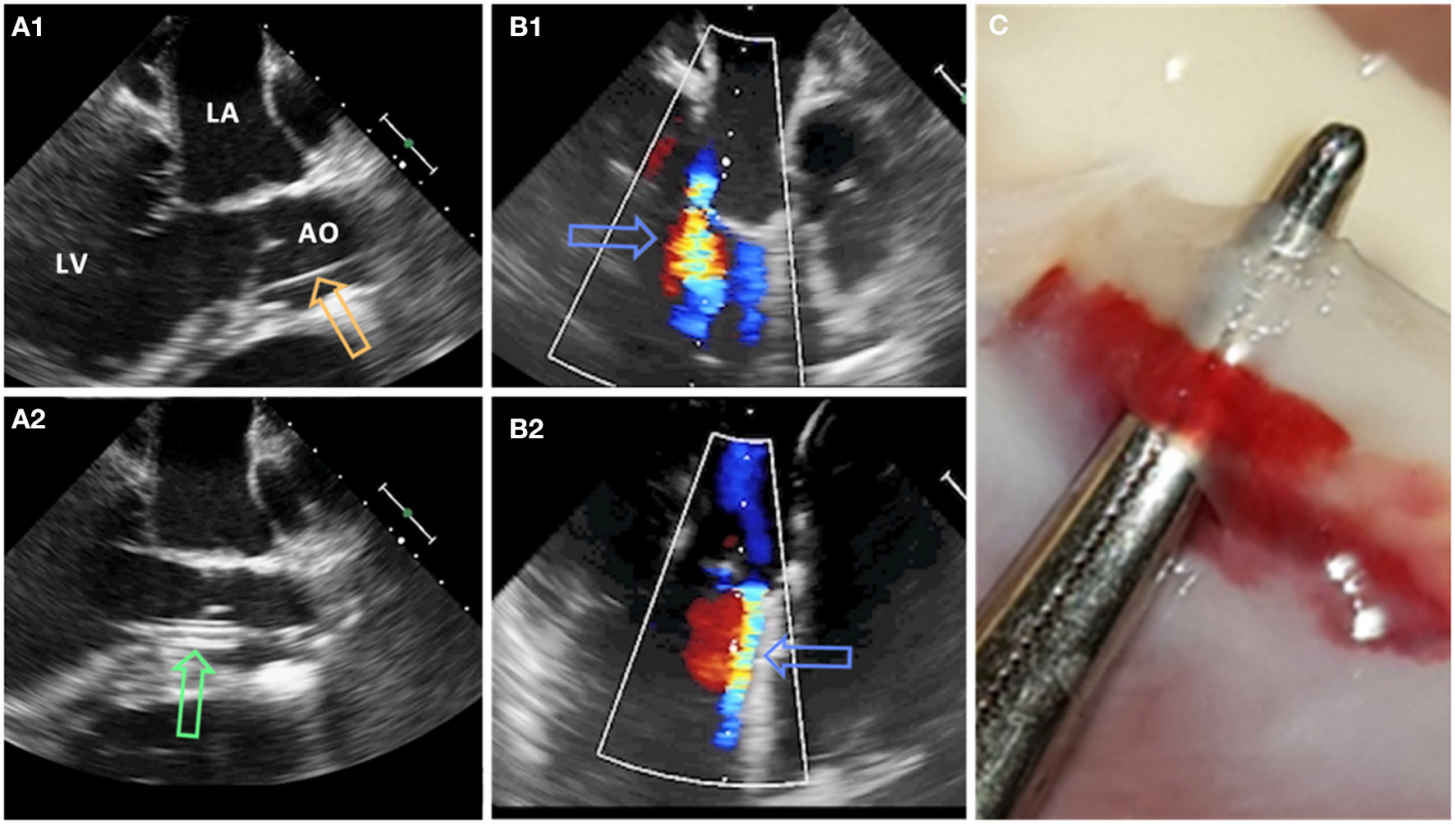
Pierce and dilate technique used to create the model of mild eccentric aortic regurgitation/para valvular leakage (AO, aorta; LA, left atrium; LV, left ventricle). **(A1)** Coronary guide wire (orange arrow) is used to pierce the leaflet/annulus at the hinge point. **(A2)** Standard PTCA 5 mm balloon (green arrow) is positioned across the defect and inflated. **(B1,B2)** Aortic regurgitant jet (blue arrows) originating from NCC and RCC region, respectively. **(C)** Defect of the aortic valve at the hinge point of the leaflet.

At the beginning of the experiment, all animals received premedication with ketamine (20 mg/kg), azaperone (1.5 mg/kg), and atropine (0.75 mg) intramuscularly. After loss of postural reflexes, the anesthesia was deepened by a bolus injection of propofol (1–2 mg/kg) to facilitate intubation. Anesthesia maintained with 2–3% isoflurane and propofol (2–5 mg/kg/h) for the remainder of the experiment. Amiodarone (2–3 mg/kg bolus iv) was administered to stabilize the heart rhythm. Pain management included fentanyl infusion (0.02 mg/kg/h) for the duration of the procedure. Aortic valve defects were induced with transcatheter approach by a pierce and dilate technique at the hinge points of the NCC or the RCC, respectively. Due to its symmetrical position as NCC in respect to the mitral valve, close proximity of the orifice of the left coronary artery and potential life threatening complications if partially occluded during the intervention, left coronary cusp (LCC) defect was omitted in this study. In brief, the target point in the NCC or RCC was reached under Echo and Fluoroscopy guidance by using a steerable sheet (Agilis™ EPI Steerable Sheath, 8.5F, St. Jude Medical, Minnetonka, MN, USA). The annulus/leaflet hinge was pierced with a stiff-end of a coronary guide-wire (IRON MAN Guide Wire, 0.014″ 190 cm, Abbott Vascular, Santa Clara CA, USA). The puncture was then dilated with a 5 mm PTCA balloon (NC Emerge MONORAIL™ PTCA Dilation Balloon 5 x 12 mm, Boston Scientific Corporation, Marlborough, MA, USA) (Figure 1). Due to the elasticity of non-calcified annular and leaflet tissue, defects have been expected to reduce the size uniformly in all animals. Hence, the effective regurgitant orifice was expected to be smaller than the 5 mm balloon used for dilatation.

After echocardiographic confirmation of the aortic valve defect, animals were transferred to the in-house MR facility where anesthesia was maintained by mechanical ventilation with 2–3% of isoflurane in a 1:1 oxygen/air mixture (4–5 L/min) for the duration of the imaging procedure. On the completion of the study procedure (~6–8 h long), all animals were euthanized by the administration of an overdose of pentobarbital while still under general anesthesia according to the animal study protocol.

### Imaging Protocol

All experiments were performed on a 3 T Philips Ingenia (Philips Healthcare, Best, The Netherlands) system equipped with a 28-channel receiver coil. The imaging protocol was adopted from (21) and in brief consisted firstly of clinical cine sequences for acquisition of the anatomical reference and functional imaging in four-chamber, two chamber, and short axis orientation: field of view (FOV) 281–360 × 198–291 mm^2^, slice thickness 8 mm, in-plane resolution 1.9 × 1.9 mm^2^, echo time (TE) 1.3–1.4 ms, repetition time (TR) 2.6–2.8 ms, flip angle (FA) 45°, cardiac phases 60. Secondly, 4D-flow MRI with 40 to 50 slices covering the whole heart in four-chamber orientation was acquired: spatial resolution of 2.6 × 2.6 × 2.6 mm^3^, FOV of 300 × 197–237 × 80–100 mm^3^, flip angle of 7°, TE/TR 2.2–2.3 ms/4.6–4.7 ms, and an isotropic encoding velocity of 160 cm/s. The acquisition was synchronized with the electrocardiogram (ECG) or pulse-oximetry signal, and was retrospectively binned to 18–25 heart phases. Imaging was performed during ventilated breathing, with a total scan duration of ~45 min.

Data were reconstructed and corrected for concomitant fields using the MRecon software package (GyroTools, Winthertur, Switzerland). Linear eddy-current-induced background phase errors were corrected for by referencing through stationary tissue (22) implemented in GTFlow (GyroTools, Winthertur, Switzerland).

### Data Analysis

**Figure 2:**
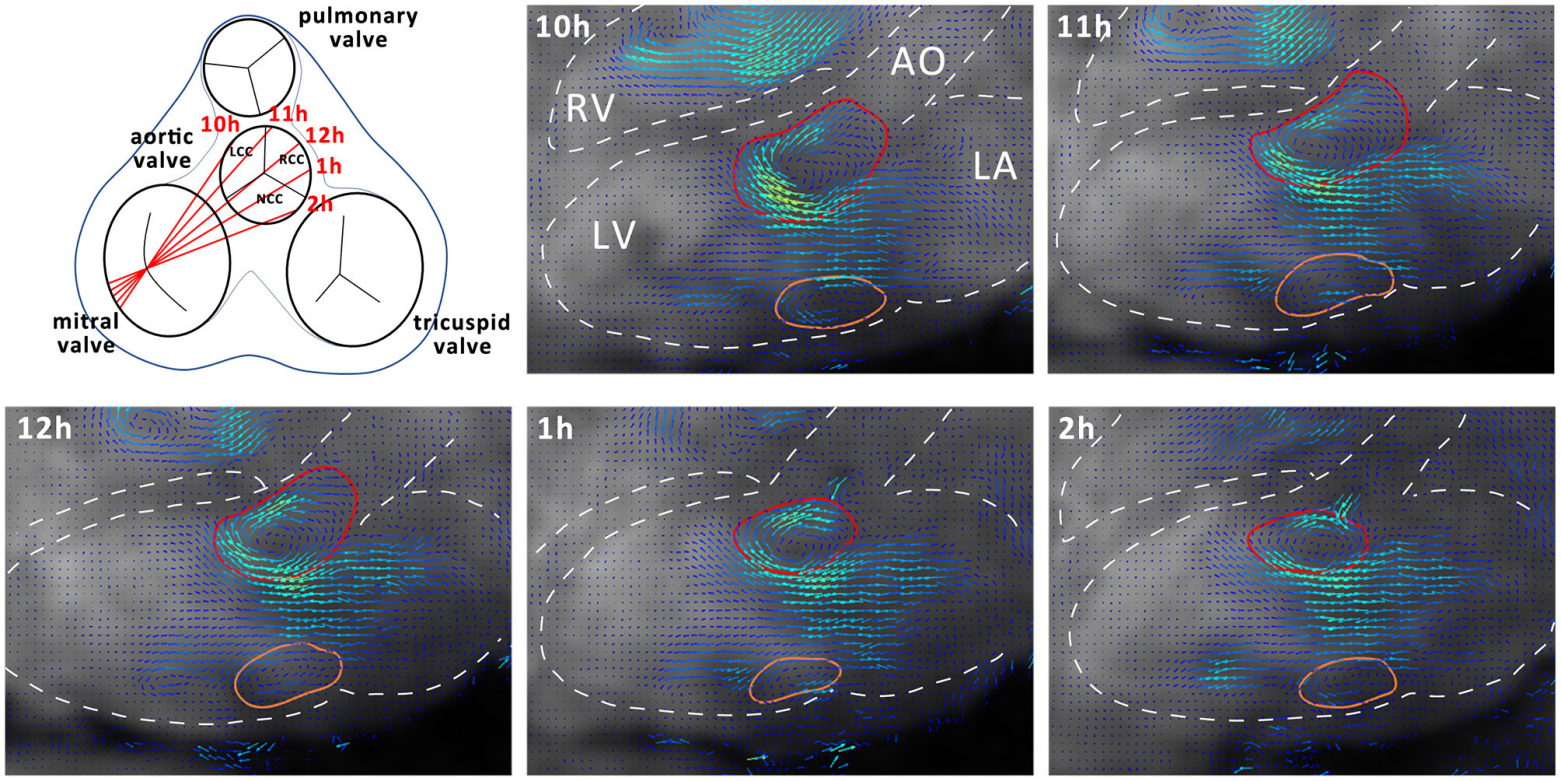
Schematic representation of different observation planes annotated as 10–2 h and example images for vortex cross sectional area estimation. Dashed white lines represent outline of anatomical structures of interest. AO, Aorta; LA, Left Atrium; LV, Left Ventricle; RV, Right Ventricle. Anterior cross section of the vortex is delineated in red and the posterior in orange.

Data analysis was performed using GTFlow (GyroTools, Winthertur, Switzerland). Four-dimensional flow MRI data were reformatted to identify the mitral valve c in short axis view. In early diastole a region of interest (ROI) was placed by manually drawing a contour at the inner edge of the valve. For vortex analysis, the three-chamber slice orientation was chosen, as it halves the anterior and posterior leaflets of the mitral valve, providing a good view for measuring vortex ring dimensions. For standardization, the three-chamber orientation was aligned to be a long axis view of the heart, including the left ventricular outflow tract showing the branch of the right coronary artery. Mitral valve inflow velocities averages across the mitral valve ROI were measured at defined time points during diastole (TP1, E-wave acceleration; TP2, peak E-wave; TP3, E-wave deceleration; TP4, diastasis; TP5, A-wave acceleration; TP6, peak A-wave; TP7, A-wave deceleration phase) (21). Blood flow through the left ventricle was visualized using two-dimensional vector field representations in the three-chamber orientation and three-dimensional streamline and path line calculations. By streamline tracking of NCC and RCC jets, the interaction with the mitral valve inflow and diastolic vortices was visualized. To evaluate the effects of different aortic regurgitant jet trajectories on the diastolic vortex, four additional long-axis observational planes were defined as illustrated in Figure 2. These long-axis observational cut planes were prescribed rotating the three chamber view around the long axis defined by the center point of the mitral valve in short axis view and the apex covering the width of the aortic valve from the left fibrous trigone to the right fibrous trigone. In this orientation, the 3-dimensional vortex ring was presented as two separate vortices forming behind the anterior and posterior leaflets of the mitral valve (Figure 2). Cross-sectional dimensions of the anterior and posterior vortex defined as the largest closed loop of non-zero velocities were measured in each animal. Additionally, the vortex index, defined as the Euclidean norm of the curl of the velocity vector field at each voxel (23) averaged over the vortex cross-sections, was computed and vortex development/change was tracked during E- and A-wave in diastole. Using particle trace counts initialized at the orifice of the PVL jet over diastole, the fraction of the regurgitant blood volume ejected from the left ventricle during the next R-R interval referred to as direct flow was compared for both NCC and RCC PVL groups. Particle tracking analysis failed in one animal in the RCC group.

Ventricular lumen, mitral, and aortic planes were manually segmented at the end systolic frame and tracked over the cardiac cycle using finite element image registration on short axis cine images (24). The tracked mesh was consequently used to mask 4D flow data. The specific energy per voxel was computed as $\frac{1}{2}\rho u^{2}\;$with ρ being the blood density: 1.05 g/cm^3^ and *u* the voxel velocity magnitude. Total specific kinetic energy in the lumen was obtained by summation averaging of the specific kinetic energy of the voxels within the tracked lumen. The inflow and outflow of kinetic energy through the mitral and the aortic valves were computed from the weighted average of the specific kinetic energy of the voxels within the corresponding valve planes.

### Statistic

Data on mitral inflow, aortic stroke volume, and regurgitant volume as well as cardiac output and heart rate were reported as mean and min/max values. All data on vortex analysis were presented as mean values and corresponding standard deviation across the individual cohort.

## Results

### Transcatheter Mild Aortic Regurgitation/PVL Model Creation—Procedural Success 93%

Procedure of model creation (i.e., mild eccentric aortic regurgitation/PVL originating from either NCC or RCC annular region) was successful in 93% of study animals. In one animal (NCC group), a severe regurgitation was introduced and the animal was excluded from the study. Heart rate, mitral inflow, as well as aortic stroke volume and cardiac output were comparable between the groups and averaged at 68 bpm, 64 ml, 71 ml, and 4.8 L/min, respectively (Table 1). With a regurgitant fraction of 14% for both NCC and RCC the assessment of the aortic regurgitant volume demonstrated a mild aortic regurgitation as defined by the European Association of Echocardiography (25). However, considering VARC 3 criteria, such regurgitation (RF 14%) would be regarded as trace (26). There was no significant difference between NCC, RCC, and control groups in any parameter. Peak inflow velocities averaged across the mitral valve orifice at peak E-wave (i.e., TP2) were 30 ± 7 cm/s and 33 ± 12 cm/s for the NCC and RCC group, respectively. Mitral inflow velocities were not influenced by the origin and trajectory of the mild aortic regurgitant jet and were comparable to controls at all time points during diastole (NCC/RCC/control: TP1: 19.6 ± 6/20.8 ± 8/20.5 ± 5 cm/s; TP2: 30.6 ± 7/33.8 ± 12 30.5 ± 2 cm/s; TP3 18.7 ± 5/22.7 ± 7/20.8 ± 9 cm/s; TP4: 0.8 ± 2/0.8 ± $\frac{1}{4}$ ± 6 cm/s; TP5: 14.6 ± 7/10.7 ± 4/14.3 ± 4 cm/s; TP6: 19.4 ± 2/19.5 ± 13/19.2 ± 1 cm/s; TP7: 4.5 ± 3/1.8 ± 4/4.5 ± 7 cm/s).

**Table 1:** Hemodynamic values of animals used.

**Group**	***N***	**Heart rate (bpm)**	**Mitral inflow volume (ml/stroke)**	**Aortic stroke volume (ml/stroke)**	**Cardiac output (L/min)**	**Regurgitant volume (ml/stroke)**	**Regurgitant fraction %**
NCC anterior	5	66 (60–78)	66 (61–80)	71 (55–88)	4.7 (3.7–5.3)	10 (8–14)	14 (10–18)
RCC posterior	6	69 (66–75)	59 (51–66)	71 (61–78)	4.8 (4.2–5.5)	10 (8–13)	14 (11–17)
Control	2	69 (66–71)	74 (69–80)	72 (69–74)	4.8	-	-

*No statistically significant difference in heart rate, aortic stroke, and mitral inflow volume as well as in cardiac output could be observed between any of the study groups. PVL/AI regurgitant volume and regurgitant fraction were comparable between the NCC and RCC group*.

### NCC Jet Did, but RCC Jet Did Not Integrate into the Diastolic Vortex

**Figure 3:**
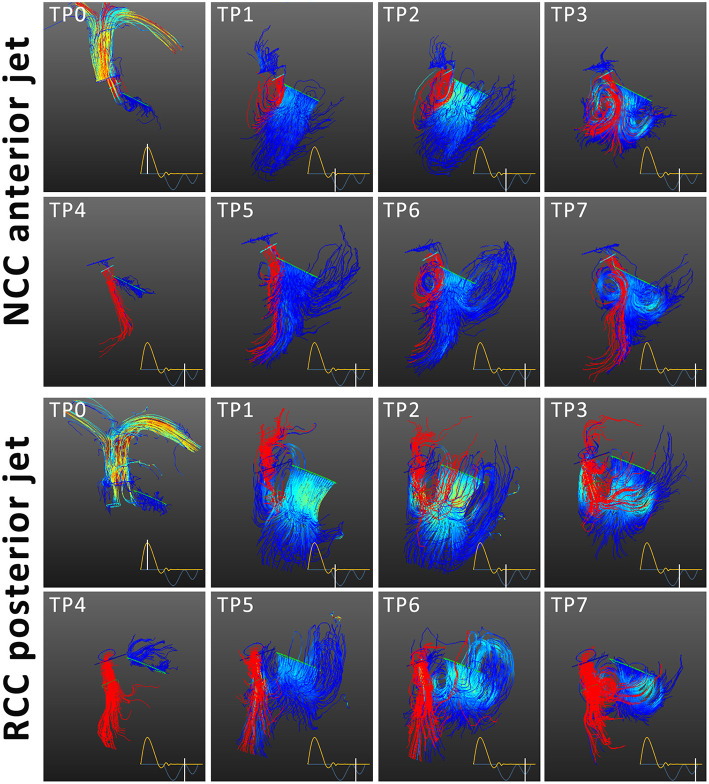
Streamline tracing of blood flow for NCC and RCC paravalvular leakage. Mitral inflow in blue and aortic regurgitant jet in red. The time points correspond to ejection (TP0), E-wave acceleration (TP1) peak E-wave (TP2) E-wave deceleration (TP3), diastasis (TP4), A-wave acceleration (TP5), peak A-wave (TP6), A-wave deceleration phase (TP7). The mitral and aortic streamlines are color coded according to the flow velocities. The regurgitant jet is depicted in red. The NCC jet integrated into the anterior vortex and the mitral inflow jet, while the RCC jet is directed along the septum and collides with the anterior vortex during filling.

Streamline tracking (Figure 3) revealed that the NCC regurgitant jet got predominantly integrated into the diastolic vortex forming in the left ventricular outflow track and the inflow jet of the mitral valve during the filling phase (E and A phase). Consequently the regurgitant blood was largely contained within the basal segments of the LV. In contrast, the RCC jets were directed along the septum and collided with the anterior vortex in the area of opposing flow direction during both filling phases (E and A phase). During diastasis, the RCC jets reached to the apical region of the LV (Figure 3).

### RCC Jet Negatively Influenced Timely Development of the Vortex

Additionally, the presence of eccentric mild aortic regurgitation/PVL was found to negatively affect the temporal development of the vortex. A distinct reduction of vortex presence during E-wave was found for the jets originating from RCC, while for the NCC jet the vortex was visible for all timepoints during E-wave except one case during E-wave acceleration (Table 2) in the three-chamber view cut plane. Mild aortic regurgitation/PVL induced absence of anterior vortex formation was also found during early A-wave, where 20% of NCC and 0% RCC animals displayed a vortex in the LVOT. The vortex was visible in all control subjects at that timepoint (Table 2). During peak A-wave and A-wave deceleration phase, the vortex was visible in all subjects. The presence of the vortex behind the posterior leaflet of the mitral valve was comparable for all animals in this study.

**Table 2:** Presence of anterior and posterior vortex for each time point (TP) at the site of PVL jet impact.

	**Group**	**TP1 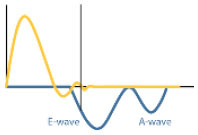 **	**TP2 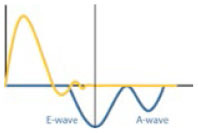 **	**TP3 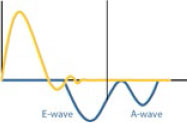 **	**TP4 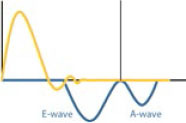 **	**TP5 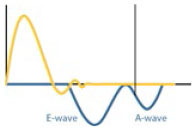 **	**TP6 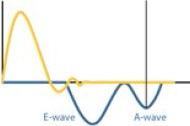 **	**TP7 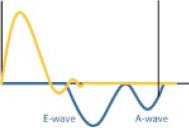 **
Anterior	NCC	4/5	5/5	5/5	0/5	1/5	5/5	5/5
vortex	RCC	3/6	4/6	4/6	1/6	0/6	6/6	6/6
	Control	2/2	2/2	2/2	1/2	2/2	2/2	2/2
Posterior	NCC	5/5	5/5	5/5	0/5	2/5	5/5	5/5
vortex	RCC	5/6	5/6	5/6	0/6	1/6	5/6	4/6
	Control	2/2	2/2	2/2	1/2	2/2	2/2	2/2

*Data presented as fraction of animals where vortex was observed and all animals in the corresponding group. TP1, E-wave acceleration; TP2, peak E-wave; TP3, E-wave deceleration; TP4, diastasis; TP5, A-wave acceleration; TP6, peak A-wave; TP7, A-wave deceleration phase*.

### RCC Jet Locally Induced Reduction in Vortex Size

**Figure 4:**
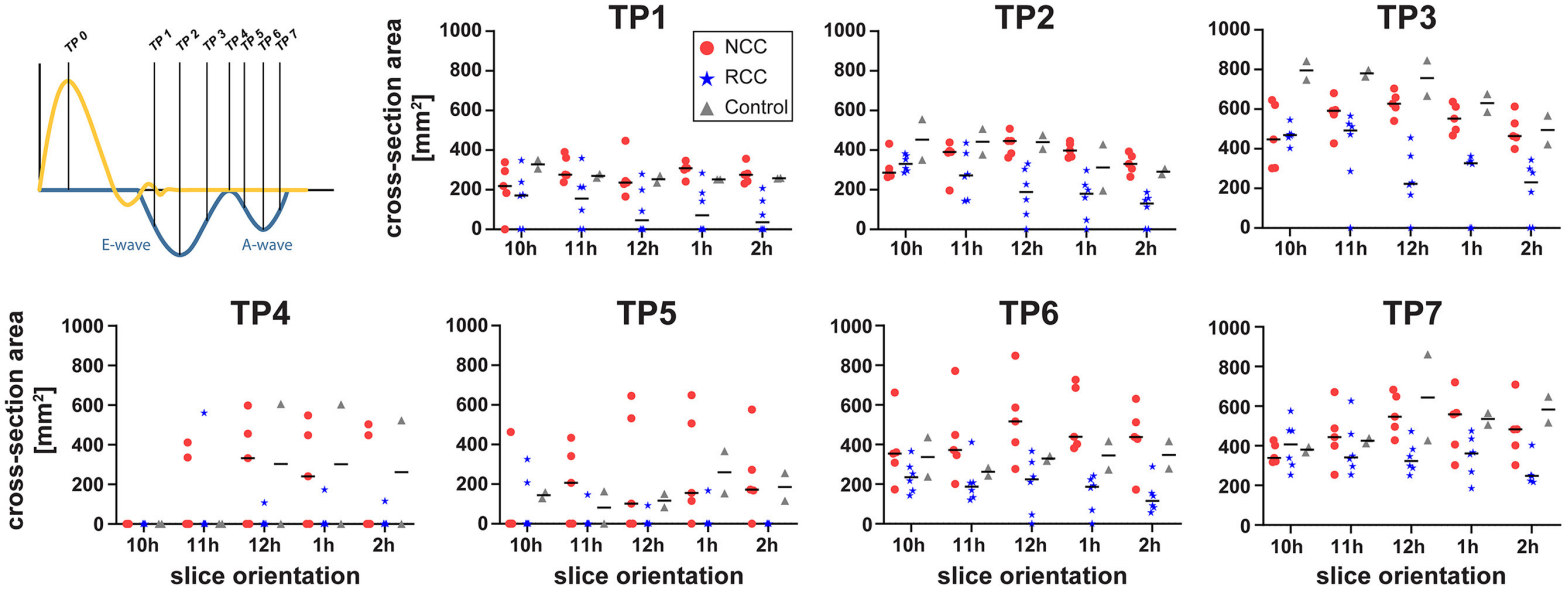
Cross-sectional area of the anterior vortex for different long axis cut planes (according to Figure 2) for NCC (red circles), RCC (blue stars), and control (grey triangles) animals.

By using multiple cut planes as described in Figure 2, assessment of vortex cross sectional areas was performed. In control animals the cross-sectional size of the diastolic vortex was comparable between the observation cut planes at the same heart phase (Figure 4). Mild aortic regurgitant jets tended to affect the parts of the vortex ring differently depending on the jets' origins. In the NCC group the vortex size as function of cut plane positions followed the trend of the control animals. The largest cross-sectional area of the anterior diastolic vortex in the direct path of the PVL jet (NCC) was observed during the E-Wave deceleration phase (TP3), which amounted to an average across all NCC animals of 573 mm^2^ (range 253–680 mm^2^). In contrast, the anterior vortex size was found to be on average 183 mm^2^ (range 0–344 mm^2^) in the RCC group during the same heart phase. It was noted that the reduction of vortex cross sectional area in the RCC group was found to be localized predominantly in the cut planes around 12 and 1 h as defined in Figure 2. For the area of the posterior vortex no differences were found across groups. No differences in average and maximal vorticity index between NCC and RCC PVL animals were found when a vortex was still present.

### RCC Regurgitant Blood Displayed Prolonged LV Residence Time

**Figure 5:**
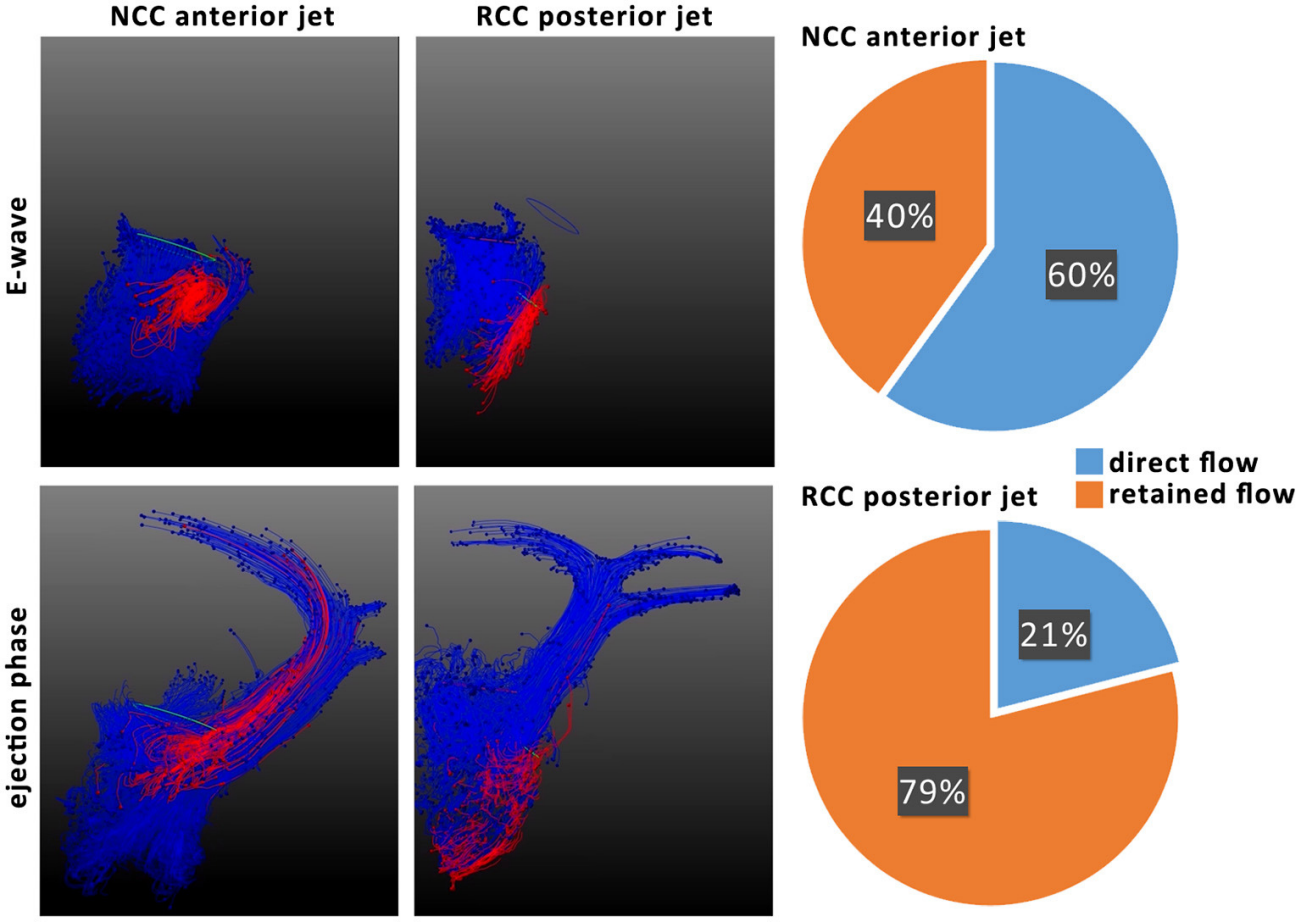
Particle tracking of NCC and RCC regurgitant jets to illustrate residence time of regurgitant blood (left). Particles originating from mitral valve inflow are colored in blue and particles from the regurgitant jet red. Ratios of direct and retained flow of the regurgitant blood volume are shown on the right. Direct flow is the part of diastolic inflowing blood volume that is ejected in the next systole, whereas retained flow represents the diastolic inflow volume that resides in the ventricle for more than 1 heart beat.

The origin of the mild eccentric regurgitant/PVL jet (either NCC or RCC) and its trajectory within the left ventricle had a profound effect on the residence of the regurgitant blood within the LV lumen across consecutive heart beats. Whereas most of the NCC regurgitant blood volume remained in the basal segments of the LV, RCC regurgitant blood reached further to the apex of the LV and remained within the ventricle during the next ejection phase. Subsequently, the direct flow fraction was calculated to be on average 60% (43–76%) for all NCC subjects and 21% (12–33%) for all RCC subjects (Figure 5). Particle tracking illustrated the mixing of the NCC regurgitant blood with the mitral inflow and ejection from the LV cavity in the consecutive heart beat (Figure 5).

#### Global Left Ventricular Kinetic Energy Was Not Influenced by Mild Aortic PVL Jet

No major difference in specific kinetic energy measured in the LV cavity was found between NCC, RCC, and control group. The specific kinetic energy within the cavity was dominated by the kinetic energy influx through the mitral valve. Concerning the specific kinetic energy flux from the PVL jet, both NCC and RCC jets carried similar specific kinetic energies: TP1: 46.7 ± 35.6/40.6 ± 41.6 J/m^3^, TP2: 43.8 ± 20.8/42.3 ± 49.5 J/m^3^, TP3: 34.9 ± 16.7/30.1 ± 20.3 J/m^3^, TP4: 15.5 ± 8.0/11.6 ± 7.4 J/m^3^, TP5: 15.0 ± 5.8/10.9 ± 14.5 J/m^3^, TP6: 17.5 ± 8.0/9.6 ± 9.1 J/m^3^, and TP7: 13.2 ± 4.7/9.2 ± 7.5 J/m^3^. The largest backflow of kinetic energy through the orifice of the PVL was found during early and peak E-wave.

## Discussion

In this work we present a novel, minimally invasive (transcatheter), translational large animal model of mild eccentric aortic regurgitation/PVL—a common complication after TAVR procedure. With the developed model, we are able to simulate PVLs originating from the NCC or the RCC annular region, with a procedural success rate of 93%. The cardiac MRI assessment of regurgitant volume, regurgitant fraction, and back flux of kinetic energy by the PVL jets showed no differences between the NCC and RCC groups, demonstrating a large degree of reproducibility across animals even when targeting different leaflets of the aortic valve.

Although there seems to be some misalignment on the classification of the PVL/AR in past studies and current guide lines (2, 25, 26), regurgitation induced in our study (14% regurgitant fraction) can be described as quite discrete (mild or trace depending on classification). PVLs of similar intensity have been found to have no marked influence on long term mortality in clinical trials (2). Still, PVL/AR jets investigated in our study induced local disturbance of vortex formation. However, portions of the vortex ring medial or lateral from the point of jet-impact were only marginally affected and remained comparable to controls, further demonstrating the discrete nature of the induced changes. By analyzing the acquired velocity vector fields, we could show that NCC jets oriented along the ventricular side of the anterior mitral leaflet got integrated in the naturally occurring diastolic vortex in LVOT. Consequently, NCC jets only marginally affected the vortex size. On the other hand, RCC jets were directed along the septal wall and locally reduced the cross-sectional size of the vortex delaying or even prohibiting vortex formation. Reduced vortex size and delayed vortex formation time was observed in patients and confirmed in models simulating left ventricular hypertrophy (27, 28). These findings were attributed to the reduced LV volume not providing sufficient space for proper vortex development, ultimately resulting in increased kinetic energy dissipation (27, 28). Hence, extrapolating these findings to our study of healthy porcine hearts, it could be argued that RCC regurgitant jets induced a “fluid dynamic” correlate to reduction of space available for proper diastolic vortex development in hypertrophic left ventricle. Translated to TAVR patients with ventricular hypertrophy, such unfavorable PVL jet orientation could display additive effects of anatomical and “fluid dynamic” factors effectively leading to even more pronounced blood flow disturbance and kinetic energy dissipation.

As a direct consequence of mild aortic regurgitant/PVL jet origin and trajectory, regurgitant blood originating from NCC PVL remained mostly in the basal area of the LV and was ejected during the consequent heart beat, whereas RCC jets were reaching the apical sections of the LV leaving, to a large extent, the regurgitant blood in the LV during the subsequent contraction. It has been shown previously that direct passage of blood within a single heart beat is associated with preservation of inflow kinetic energy, whilst prolonged durations of blood remaining within the cavity is associated with reduced preservation of kinetic energy (29). In diseases affecting the entire heart such as dilated cardiomyopathy, a significant increase in the retained flow fraction was found (29, 30). It has been shown that despite having a lower fraction of direct flow the specific kinetic energy of the direct flow component was similar to healthy controls. In contrast, the specific kinetic energy of all remaining flow components was elevated in the DCM cohort. The authors hypothesize that changes in specific kinetic energies may alter the interaction of blood flow and myocardial wall. In our study we investigated the localized phenomenon of PVL. We could not find differences in kinetic energies within the left ventricular blood pool, however a significant increase of retained regurgitant blood volume for RCC PVLs together with a local alteration of vortex formation. This effect may chronically impair the interaction of ventricular wall and blood flow and may ultimately stimulate a remodeling process to provide an optimal geometry for efficient flow (15, 31).

*In-silico* (simulator) studies showed spatial dependency of PVL jets interaction with the ventricular vortex formation and its effects on kinetic energy (18). However, in this occasion severe aortic PVLs with regurgitant fraction of 41% were studied. In contrast clinical, pre-clinical, and simulator studies of central (or indiscriminate) aortic regurgitation could demonstrate that even mild regurgitant jets can cause relevant disturbances for diastolic flow patterns and increase energy dissipation (17, 32, 33). Despite providing valuable insights, such studies were limited to evaluations of hemodynamic effects in a single plain corresponding to PVL jet trajectory. In our study the use of 4D flow, MRI allowed for re-slicing data along the vortex ring showing that the inhibiting effect of mild eccentric aortic regurgitant/PVL jets on vortex formation does not degrade the entire vortex ring. In accordance with the simulator work we found that the RCC location of PVLs alters the ventricular hemodynamics significantly while NCC PVLs do not, for mild leakages. With the translational model presented here, it is possible to induce different scenarios of vortex formation which can be used to investigate the impact of blood flow disturbances on the ventricle. For example, disturbed vortices have been found in patients suffering from heart failure, LV hypertrophy, or after valvular interventions (29, 34, 35). However, the causative vortex-ventricle relations are not yet fully understood.

In the clinical treatment of PVLs great care is given to the regurgitant volume as therapy thresh-hold and predictor of outcome. Translational data shown here suggest caution when dealing with less-than-severe aortic PVLs. Results presented in this study demonstrate that even mild aortic PVL depending on their origin and flow directionality (i.e., more pronounced in RCC than in NCC jets) can adversely impact LV hemodynamics. Although premature to make statements regarding need for treatment of less-than-severe PVLs, translational insights from this study suggest that analysis of jet origin and trajectory should be investigated in a longitudinal study to assess potential for risk stratification in patients suffering from aortic PVLs.

Although there are some anatomical and physiological differences, porcine cardiac structures and hemodynamic parameters closely resemble that of humans. The translational large animal model of mild eccentric aortic regurgitation/PVL used in this study allows MRI based investigation of blood flow patterns under controlled and reproducible conditions (i.e., general anesthesia, controlled ventilation, heart rate, and blood pressure). PVLs with standardized regurgitant flow, fraction, and orifice area, induced in a trans-catheter fashion in a native aortic valve of healthy pigs, further helped isolate the effects of regurgitant flow without restraining factors such as comorbidities. The proposed animal model (i.e., without TAVR implant) also greatly facilitates imaging of PVLs as implanted prosthetic valve scaffolding could lead to severe off-resonance artifacts when imaged by MRI. Resulting signal voids appear not only at the site of implantation but also reach into the LVOT. As a consequence, it is very challenging to image PVL jets at their origin in the proximity of the implant by 4D flow MRI. Furthermore, without major manipulations of the TAVR implant itself, it would be challenging to obtain reproducible PVLs (if any) in the healthy porcine model. On the other hand, imaging performed on human-grade scanners ultimately facilitates the translation of study results into patients. Hence, the animal study presented here provides translational insights on effects of mild aortic PVL, for a patient population that would be, otherwise, challenging to investigate.

### Study Limitations

This study was performed under general anesthesia, which has an effect on heart rate as well as on blood pressure, consequently affecting the afterload of the heart. Changes in systemic blood pressure may alter regurgitant volume and jet velocities altering the effect of PVL jets on ventricular blood flow patterns. In the *in-vivo* animal model, these physiological parameters may be controlled pharmaceutically. A less challenging control of environmental parameters has been proposed in isolated beating pig hearts with good data quality and reproducibility (20). Further, only healthy animals without prior aortic valve stenosis and respective left ventricular hypertrophy have been used in this study. Hence the effects of prior disease as it would be observed in clinical patients receiving TAVR as well as PVL jets impact on flow patterns within a hypertrophic left ventricle could not be reflected in our study and should be mirrored in future trials. Moreover, invasive hemodynamic assessment (e.g., pressure-volume loop analysis) could have provided more insight of effects of volume overload caused by mild AI/PVL, especially as no differences in kinetic energy were observed between the groups in our study. As they were not implemented in the current trial, such measurements should be considered going forward.

### Conclusion

We have established a translational large animal model to create PVLs with a transcatheter technique and assessed their impact on left ventricular hemodynamics by 4D flow MRI.

The origin and trajectory of otherwise comparable mild aortic PVL jets induced different effects on diastolic LV hemodynamics. While regurgitant blood originating from NCC PVL jets got well integrated into the diastolic vortex and was largely contained within the LVOT, RCC jets firstly collided with the vortex, markedly reducing its size, and finally reached the apical region of LV where it resided over the consequent heartbeat.

These translational results suggest that investigating origin and trajectory of mild aortic PVL jets may provide additional information for decision making regarding interventions and potentially play a key role for long-term remodeling in affected patients.

## Data Availability Statement

The raw data supporting the conclusions of this article will be made available by the authors, without undue reservation.

## Ethics Statement

The animal study was reviewed and approved by Zurich cantonal Veterinary Office.

## Author Contributions

NC: conceptualization, study design, development of animal model, surgical procedure, animal handling, data acquisition, data analysis, statistical analysis, and preparation and review of manuscript. MW, MK, and MC: development of animal model, surgical procedure, and animal handling. LG, EP, and SB: data analysis and review of manuscript. VF, SS, SK, and ME: data interpretation and critical review of the manuscript. CS: conceptualization, study design, data acquisition, data reconstruction, data analysis, and preparation and review of manuscript. All authors contributed to the article and approved the submitted version.

## Conflict of Interest

VF has relevant (institutional) financial activities outside the submitted work with following commercial entities: Medtronic GmbH, Biotronik SE & Co., Abbott GmbH & Co. KG, Boston Scientific, Edwards Lifesciences, Berlin Heart, Novartis Pharma GmbH, JOTEC/CryoLife GmbH, Zurich Heart. The remaining authors declare that the research was conducted in the absence of any commercial or financial relationships that could be construed as a potential conflict of interest.

## Publisher's Note

All claims expressed in this article are solely those of the authors and do not necessarily represent those of their affiliated organizations, or those of the publisher, the editors and the reviewers. Any product that may be evaluated in this article, or claim that may be made by its manufacturer, is not guaranteed or endorsed by the publisher.

## References

[1] Dvir D Barbash IM Ben-Dor I Torguson R Badr S Minha S. Paravalvular regurgitation after transcatheter aortic valve replacement: diagnosis, clinical outcome, preventive and therapeutic strategies. Cardiovasc Revasc Med. (2013) 14:174–81. 10.1016/j.carrev.2013.02.003 23773501

[2] Ribeiro HB Orwat S Hayek SS Larose E Babaliaros V Dahou A. Cardiovascular magnetic resonance to evaluate aortic regurgitation after transcatheter aortic valve replacement. J Am Coll Cardiol. (2016) 68:577–lpage>85. 10.1016/j.jacc.2016.05.059 27491900

[3] Kodali S Pibarot P Douglas PS Williams M Xu K Thourani V. Paravalvular regurgitation after transcatheter aortic valve replacement with the Edwards sapien valve in the PARTNER trial: characterizing patients and impact on outcomes. Eur Heart J. (2015) 36:449–lpage>56. 10.1093/eurheartj/ehu384 25273886

[4] Hayashida K Lefevre T Chevalier B Hovasse T Romano M Garot P. Impact of post-procedural aortic regurgitation on mortality after transcatheter aortic valve implantation. JACC Cardiovasc Interv. (2012) 5:1247–lpage>56. 10.1016/j.jcin.2012.09.003 23257373

[5] Kampaktsis PN Subramayam P Sherifi I Vavuranakis M Siasos G Tousoulis D. Impact of paravalvular leak on left ventricular remodeling and global longitudinal strain 1 year after transcatheter aortic valve replacement. Future Cardiol. (2021) 17:337–lpage>45. 10.2217/fca-2020-0086 33590775

[6] Tang GHL Zaid S Fuchs A Yamabe T Yazdchi F Gupta E. Alignment of Transcatheter Aortic-Valve Neo-Commissures (ALIGN TAVR): impact on final valve orientation and coronary artery overlap. JACC Cardiovasc Interv. (2020) 13:1030–lpage>42. 10.1016/j.jcin.2020.02.005 32192985

[7] Mollmann H Holzhey DM Hilker M Toggweiler S Schafer U Treede H. The ACURATE neo2 valve system for transcatheter aortic valve implantation: 30-day and 1-year outcomes. Clin Res Cardiol. (2021). 10.1007/s00392-021-01882-3. [Epub ahead of print]. 34148125 PMC8639565

[8] Popma JJ Deeb GM Yakubov SJ Mumtaz M Gada H O'Hair D. Transcatheter aortic-valve replacement with a self-expanding valve in low-risk patients. N Engl J Med. (2019) 380:1706–lpage>15. 10.1056/NEJMoa1816885 30883053

[9] Baumgartner H Falk V Bax JJ De Bonis M Hamm C Holm PJ. 2017 ESC/EACTS Guidelines for the management of valvular heart disease. Eur Heart J. (2017) 38:2739–lpage>91. 10.1093/eurheartj/ehx391 28886619

[10] Jones BM Tuzcu EM Krishnaswamy A Popovic Z Mick S Roselli EE. Prognostic significance of mild aortic regurgitation in predicting mortality after transcatheter aortic valve replacement. J Thorac Cardiovasc Surg. (2016) 152:783–lpage>90. 10.1016/j.jtcvs.2016.05.023 27321435

[11] Kampaktsis PN Ullal AV Minutello RM Feldman DN Swaminathan RV Voudris K. Impact of paravalvular aortic insufficiency on left ventricular remodeling and mortality after transcatheter aortic valve replacement. J Heart Valve Dis. (2016) 25:301–lpage>8. 27989040

[12] Arvidsson PM Kovacs SJ Toger J Borgquist R Heiberg E Carlsson M. Vortex ring behavior provides the epigenetic blueprint for the human heart. Sci Rep. (2016) 6:22021. 10.1038/srep22021 26915473 PMC4768103

[13] Kanski M Arvidsson PM Toger J Borgquist R Heiberg E Carlsson M. Left ventricular fluid kinetic energy time curves in heart failure from cardiovascular magnetic resonance 4D flow data. J Cardiovasc Magn Reson. (2015) 17:111. 10.1186/s12968-015-0211-4 26685664 PMC4685624

[14] Pedrizzetti G Sengupta PP. Vortex imaging: new information gain from tracking cardiac energy loss. Eur Heart J Cardiovasc Imaging. (2015) 16:719–lpage>20. 10.1093/ehjci/jev070 25839836

[15] Kilner PJ Yang GZ Wilkes AJ Mohiaddin RH Firmin DN Yacoub MH. Asymmetric redirection of flow through the heart. Nature. (2000) 404:759–lpage>61. 10.1038/35008075 10783888

[16] Hirasawa K Izumo M Sasaoka T Ashikaga T Suzuki K Harada T. Effect of aortic regurgitant jet direction on mitral valve leaflet remodeling: a real-time three-dimensional transesophageal echocardiography study. Sci Rep. (2017) 7:8884. 10.1038/s41598-017-09252-8 28827606 PMC5567050

[17] Stugaard M Koriyama H Katsuki K Masuda K Asanuma T Takeda Y. Energy loss in the left ventricle obtained by vector flow mapping as a new quantitative measure of severity of aortic regurgitation: a combined experimental and clinical study. Eur Heart J Cardiovasc Imaging. (2015) 16:723–lpage>30. 10.1093/ehjci/jev035 25762562

[18] Morisawa D Falahatpisheh A Avenatti E Little SH Kheradvar A. Intraventricular vortex interaction between transmitral flow and paravalvular leak. Sci Rep. (2018) 8:15657. 10.1038/s41598-018-33648-9 30353062 PMC6199255

[19] Keshavarz-Motamed Z Khodaei S Rikhtegar Nezami F Amrute JM Lee SJ Brown J. Mixed Valvular disease following transcatheter aortic valve replacement: quantification and systematic differentiation using clinical measurements and image-based patient-specific *in silico* modeling. J Am Heart Assoc. (2020) 9:e015063. 10.1161/JAHA.119.015063 32106747 PMC7335548

[20] Peper ES Leopaldi AM van Tuijl S Coolen BF Strijkers GJ Baan J Jr. An isolated beating pig heart platform for a comprehensive evaluation of intracardiac blood flow with 4D flow MRI: a feasibility study. Eur Radiol Exp. (2019) 3:40. 10.1186/s41747-019-0114-5 31650367 PMC6813403

[21] Cesarovic N Busch J Lipiski M Fuetterer M Fleischmann T Born S. Left ventricular blood flow patterns at rest and under dobutamine stress in healthy pigs. NMR Biomed. (2019) 32:e4022. 10.1002/nbm.4022 30403426

[22] Busch J Giese D Kozerke S. Image-based background phase error correction in 4D flow MRI revisited. J Magn Reson Imaging. (2017) 46:1516–lpage>25. 10.1002/jmri.25668 28225577

[23] von Spiczak J Crelier G Giese D Kozerke S Maintz D Bunck AC. Quantitative analysis of vortical blood flow in the thoracic aorta using 4D phase contrast MRI. PLoS ONE. (2015) 10:e0139025. 10.1371/journal.pone.0139025 26418327 PMC4587936

[24] Genet M Stoeck CT von Deuster C Lee LC Kozerke S. Equilibrated warping: finite element image registration with finite strain equilibrium gap regularization. Med Image Anal. (2018) 50:1–lpage>22. 10.1016/j.media.2018.07.007 30173000

[25] Lancellotti P Tribouilloy C Hagendorff A Moura L Popescu BA Agricola E. European Association of Echocardiography recommendations for the assessment of valvular regurgitation. Part 1: aortic and pulmonary regurgitation (native valve disease). Eur J Echocardiogr. (2010) 11:223–lpage>44. 10.1093/ejechocard/jeq030 20375260

[26] Varc-3 Writing C Genereux P Piazza N Alu MC Nazif T Hahn RT. Valve Academic Research Consortium 3: updated endpoint definitions for aortic valve clinical research. Eur Heart J. (2021) 42:1825–lpage>57. 10.1093/eurheartj/ehaa799 33871579

[27] Samaee M Nelsen N Gaddam M Santhanakrishnan A. Diastolic vortex alterations with reducing left ventricular volume: an *in vitro* study. J Biomech Eng. (2020). 142:121006. 10.1115/1.4047663 32601698

[28] Pagel PS Hudetz JA. Chronic pressure-overload hypertrophy attenuates vortex formation time in patients with severe aortic stenosis and preserved left ventricular systolic function undergoing aortic valve replacement. J Cardiothorac Vasc Anesth. (2013) 27:660–lpage>4. 10.1053/j.jvca.2013.01.007 23727466

[29] Eriksson J Bolger AF Ebbers T Carlhall CJ. Four-dimensional blood flow-specific markers of LV dysfunction in dilated cardiomyopathy. Eur Heart J Cardiovasc Imaging. (2013) 14:417–lpage>24. 10.1093/ehjci/jes159 22879457 PMC3626338

[30] Svalbring E Fredriksson A Eriksson J Dyverfeldt P Ebbers T Bolger AF. Altered diastolic flow patterns and kinetic energy in subtle left ventricular remodeling and dysfunction detected by 4D flow MRI. PLoS ONE. (2016) 11:e0161391. 10.1371/journal.pone.0161391 27532640 PMC4988651

[31] Richter Y Edelman ER. Cardiology is flow. Circulation. (2006) 113:2679–lpage>82. 10.1161/CIRCULATIONAHA.106.632687 16769924

[32] Okafor I Raghav V Condado JF Midha PA Kumar G Yoganathan AP. Aortic regurgitation generates a kinematic obstruction which hinders left ventricular filling. Ann Biomed Eng. (2017) 45:1305–lpage>14. 10.1007/s10439-017-1790-z 28091966

[33] Di Labbio G Kadem L. Jet collisions and vortex reversal in the human left ventricle. J Biomech. (2018) 78:155–lpage>60. 10.1016/j.jbiomech.2018.07.023 30049450

[34] Stoll VM Hess AT Rodgers CT Bissell MM Dyverfeldt P Ebbers T. Left ventricular flow analysis. Circ Cardiovasc Imaging. (2019) 12:e008130. 10.1161/CIRCIMAGING.118.008130 31109184 PMC6544522

[35] Morichi H Itatani K Yamazaki S Numata S Nakaji K Tamaki N. Influences of mitral annuloplasty on left ventricular flow dynamics assessed with 3-dimensional cine phase-contrast flow magnetic resonance imaging. J Thorac Cardiovasc Surg. (2020). 10.1016/j.jtcvs.2020.04.127. [Epub ahead of print]. 32690416

